# Effects of Immunostimulants on Physiological Performances, Immune Gene Expression, Liver, and Intestinal Protection Against *Vibrio parahaemolyticus* Infection in Juvenile Asian Seabass (*Lates calcarifer*)

**DOI:** 10.1155/anu/8855823

**Published:** 2025-07-30

**Authors:** Yun Liu, Qian-Qian Chen, Victor Charlie Andin, Wei-Kang Chor, Chou-Min Chong, Po-Tsang Lee, Crystale Siew-Ying Lim, Jiun-Yan Loh

**Affiliations:** ^1^Health Science Center, Guangxi University of Science and Technology, Liuzhou, China; ^2^Department of Oncology, The Fourth Affiliated Hospital of Guangxi Medical University, Liuzhou, China; ^3^WorldFish Center, Batu Maung, Pulau Pinang, Malaysia; ^4^Marine Programme Conservation Department, Worldwide Fund for Nature (WWF, Malaysia), Petaling Jaya, Malaysia; ^5^International Institute of Aquaculture and Aquatic Sciences (I-AQUAS), Universiti Putra Malaysia, Port Dickson, Malaysia; ^6^Department of Aquaculture, National Taiwan Ocean University, Keelung, Taiwan; ^7^Faculty of Applied Sciences, UCSI University, Cheras, Kuala Lumpur, Malaysia; ^8^Tropical Futures Institute, James Cook University (Singapore Campus), Singapore

**Keywords:** garlic, ginger, *Lates calcarifer*, palm carotene, *Vibrio parahaemolyticus*

## Abstract

The present study aims to develop a more valuable innovative aquaculture feed for the sustainable *Lates calcarifer* aquaculture industry. An experiment with a completely random design was developed with five groups containing 0% immunostimulant and 0.5% ginger, 1.0% garlic, 0.15% palm carotene alone or in combination. Each diet was randomly assigned to triplicate groups of 30 fish (10.06 ± 0.09 g) per tank. The fish were fed once daily to apparent satiation. Throughout the 56-day feeding trial, the results suggest that although no significant differences were observed among almost all experimental groups in growth performance, feed efficiency, fish composition and disease resistance, the palm carotene supplementations downregulated intestinal tumor necrosis factor alpha (*TNF-α*) and interleukin (IL)-6 expression, decreased sum scores of liver pathological changes, increased intestinal villus surface (VS) compared with the control group. Moreover, the combined treatment shows more promising immune gene expression (intestinal *IL-1* beta (*IL-1β*) was also downregulated compared to the control), liver and intestinal protection (lower sum scores of liver pathological changes and larger intestinal VS than palm carotene alone supplementation, goblet cell (GC) number per intestinal villus was also increased compared to the control) effects on *L. calcarifer* infected with *Vibrio parahaemolyticus*. Therefore, a combination of 0.5% ginger, 1.0% garlic, and 0.15% palm carotene in the diet of *L. calcarifer* can be used as an immune booster due to fishmeal substitution to obtain better immunocompetence.

## 1. Introduction

Asian seabass (*Lates calcarifer*), also commonly referred to as barramundi, is widely distributed across the Indo–Pacific and Australia. It is a highly profitable aquaculture fish because of its exceptional physiological tolerance, rapid growth, ease of maintenance, and productive qualities [1]. Based on the analysis by Future Market Insights (FMI), sales of *L. calcarifer* have experienced a 4.1% compound annual growth rate (CAGR) from 2016 to 2020. The FMI projections indicate that the worldwide market for *L. calcarifer* is projected to continue growing at a 5.5% CAGR through 2031 [2]. However, like any other cultured fish, *L. calcarifer* is affected by pathogens due to intensive culture [3]. This fish is prone to infectious diseases, such as *Vibrio parahaemolyticus*, which can cause tens of millions of dollars in annual economic losses [1].

Vibriosis, caused by pathogenic *Vibrio* species, has emerged as one of the most devastating bacterial diseases in global aquaculture. To combat this challenge, fish farmers have increasingly incorporated food additives or antibiotics into their practices over the past few decades. However, this approach constitutes a menace to the well-being of humans and the environment due to the occurrence of residues in fish bodies [4]. In addition, antimicrobial overuse and misuse are driving an increase in antimicrobial resistance (AMR), which has become a serious global health issue [5]. Instead of chemotherapeutic agents, increasing attention is being paid to the use of immunostimulants as a prophylactic measure for the prevention of diseases in aquaculture. Immunostimulants present an appealing and promising alternative to antibiotics, chemicals, and vaccines because they can activate the immune response by enhancing the activity of phagocytes and T and B cells [6]. Among others, plant-based immunostimulants have received more attention because they are cost-effective and eco-friendly. Ginger (*Zingiber officinale*, Roscoe) is one of the most widely used spices worldwide. This folk medicine is generally considered a safe herbal medicine for treating different diseases [7]. Studies have reported that the highest survival rate (SR) in *L. calcarifer* challenged with *Vibrio harveyi* [8] and in Nile tilapia (*Oreochromis niloticus*) infected with *Aeromonas hydrophila* [9] was achieved in supplemented diets containing 0.5% and 1% ginger, respectively. Another example, such as garlic (*Allium sativum*), is widely used around the world. Its functional properties have been extensively documented for their pivotal role in altering the principal risk factors associated with chronic diseases over an extended period [10]. Numerous studies and reports have consistently affirmed that garlic can efficiently eliminate pathogenic bacteria, including *Aeromonas punctata*, *Edwardsiella tarda*, *Fibrobacter intestinalis*, *Myxococcus piscicola*, *Pseudomonas fluorescens*, *Vibrio anguillarum*, and *Yersinia ruckeri*, in freshwater fish [11]. Most garlic research in aquaculture has involved garlic extracts [12], garlic powder [13], garlic peel [14], and fresh garlic [15, 16]. Among them, Irkin et al. [13] suggested that garlic powder supplementation is recommended in feeding regimens for European sea bass (*Dicentrarchus labrax*) juveniles; however, the dosage should not exceed 2%. On the other hand, Agbebi et al. [15] found that even 30% garlic inclusion in feed has no negative effect on the liver and gut of African catfish (*Clarias gariepinus*).

Like garlic, β-carotene is one of the main palm oil processing byproducts that can be used in the food, pharmaceutical, and cosmetics industries, apart from the oleochemical industries [17]. It is extracted from empty fruit bunches of oil palm, which are an abundant agricultural waste in Malaysia [18]. β-carotene, a carotenoid with no toxicity, exhibits immunomodulatory effects in both animals and humans [19]. It can be used as a natural immunostimulant to improve fish antioxidant capacity and immune status [20]. Several studies have indicated that the administration of palm carotene to mice at a dosage of 50 mg carotene per 100 g of diet can effectively prevent DNA impairment in bone marrow, reduce peripheral leukocyte counts, and enhance survival after X-ray irradiation [21]. Administration of 0.05% palm carotene through drinking water led to a significant decrease in the percentage of mice bearing tumors [22].

Our findings in a previous study [23] indicated that incorporating 10% black soldier fly larval (BSFL) meal into the diet of *L. calcarifer* can provide the best growth performance, feed efficiency, and economic returns. However, it does not significantly improve disease resistance. To optimize feed formula, the current study aimed to examine the effects of ginger, garlic, and palm carotene on physiological performances, immune gene expression, disease resistance, and morphological characteristics of juvenile *L. calcarifer* challenged with *V. parahaemolyticus*.

## 2. Materials and Methods

### 2.1. Experimental Diets and Proximate Analysis

Based on our findings in a previous study, a 10% BSFL meal in fishmeal substitution could enhance *L. calcarifer* growth performance, feed efficiency, and economic returns [23]. A completely random experimental design was developed with five groups based on 10% BSFL inclusion in the diet. Immune treatments including ginger, garlic, and palm carotene were supplemented in five isonitrogenous and isocaloric practical diets and were designated as IT0 (without any immunostimulant, serve as control), ITgi (containing 0.5% ginger), ITga (containing 1.0% garlic), ITpc (containing 0.15% palm carotene), and ITggp (containing 0.5% ginger + 1.0% garlic + 0.15% palm carotene). The BSFL meal was provided by Sentara Group (formerly known as Nutrition Technologies Sdn. Bhd.) (Johor, Malaysia), and palm carotene (the palm phytonutrient actives) manufactured by ExcelVite Sdn. Bhd. (Ipoh, Malaysia) for aquaculture. Ginger and garlic were obtained from the local market, chopped, and stirred for later use. All feed ingredients except for the above-mentioned were purchased from Nutri Vet Livestock Sdn. Bhd. (Negeri Sembilan, Malaysia). The feed raw materials were prepared in accordance with Table 1. The ingredients were well mixed, crushed, sieved, and pelleted into a feeding diet with a particle size of 1.5 mm through a feed pelletizer machine (Model 160, Guangzhou Juncheng Machinery Equipment Co. Ltd, China). The pellets were oven-dried at 55 °C for 12 h and subsequently sealed in vacuum-packed bags. Diets were stored in a dry and dark environment at room temperature until use.

The proximate composition of all diets was determined at Apical Scientific Sdn. Bhd. (Selangor, Malaysia), in accordance with the standard Association of Official Analytical Chemists (AOAC) methods. Ash, moisture, crude fat, and crude protein were analyzed using AOAC methods 942.05, 930.15, 920.39, and 988.05 (2016). Total carbohydrate was measured in accordance with MY/STP/378 based on US FDA 21 CFR101.9 Part 101 (2017). The proximate analysis results for all diets used in this study are shown in Table 1. The diets in all the treatments had a crude protein of between 33.10% and 34.65%, crude fat of between 6.70% and 7.20%, moisture of between 9.15% and 9.90%, ash of between 8.55% and 9.65%, and carbohydrate of between 39.90% and 41.60%. The above indices among all diets showed only slight variation.

### 2.2. Fish Feeding Trial

A total of 450 apparently healthy *L. calcarifer* juveniles (10.06 ± 0.09 g, 7.97 ± 0.04 cm) were purchased from F1 Aquaculture Sdn. Bhd. (Kuala Lumpur, Malaysia) and transported to the UCSI University Aquaculture Laboratory. The fish were divided into five groups at random, with each group consisting of three replicates. They were then placed into 15 round canvas tanks, each with a capacity of 300 L and accommodation for 30 fish. During the 7 days of acclimation, the fish were adequately fed with the IT0 diet. During the 56-day feeding trial, fish were manually fed to the point of visual satiation once daily (10:00) at approximately 4% of the fish's body weight (BW). The quantity of feed supplied to each experimental tank was recorded to calculate feed intake (FI) over the 56-day feeding trial. Feces were electrically siphoned out daily before morning feeding. Any remaining unconsumed feed was siphoned off after half an hour of each feeding. Every other day, one-third of the water in each tank was exchanged. Each week, water quality parameters, including salinity, dissolved oxygen (DO), water temperature, potential of hydrogen (pH), ammonia-N, and nitrite, were monitored. The salinity, water temperature, and pH were measured with a multifunctional water quality tester (C-600, Shenzhen Yieryi Technology Equipment Co. Ltd, China) and were 8.2–10.4 ppt, 28.7–30.1 °C, and 7.1–7.8,respectively. DO levels were measured using a DO analyzer (BLE-9100, Shenzhen Yieryi Technology Equipment Co. Ltd, China) and were maintained above 5.5 mg/L. Ammonia-N and nitrite were measured using a multiparameter portable colorimeter (DR900, Hach Company, America) and were consistently <0.1 and <0.01 mg/L, respectively.

### 2.3. Fish Physiological Performance and Body Composition

Following the feeding trial, the fish were subjected to a 24-h fasting period and subsequently anesthetized using tricaine methanesulfonate (MS-222) (100 mg/L) in small, aerated tanks. The number and weight of fish in each tank were recorded to calculate the SR, total weight gain (TWG), specific growth rate (SGR), and feed conversion ratio (FCR). Six fish per tank were randomly collected and killed. The fish's BW and body length (BL) were determined for condition factor (CF), viscera and liver were measured for viscerosomatic index (VSI) and hepatosomatic index (HSI), and the body was preserved at −20 °C for subsequent proximate composition determination. The following parameters were calculated to evaluate growth performance and feed efficiency [24]:

  $$\begin{array}{c} \text{SR }\left(\text{\% }\right)=\frac{\text{Final number of fish}}{\text{Initial number of fish}}\times 100, \end{array}$$

  $$\begin{array}{c} \text{CF }\left(g/{\text{cm}}^{3}\right)=\frac{\text{BW }\left(g\right)}{{\text{BL}}^{3}\left({\text{cm}}^{3}\right)}\times 100, \end{array}$$

  $$\begin{array}{c} \text{VSI }\left(\text{\% }\right)=\frac{\text{Weight of viscera }\left(g\right)}{\text{BW }\left(g\right)}\times 100, \end{array}$$

  $$\begin{array}{c} \text{HSI }\left(\text{\% }\right)=\frac{\text{Weight of liver }\left(g\right)}{\text{BW }\left(g\right)}\times 100, \end{array}$$

  $$\begin{array}{c} \text{TWG }\left(g\right)=\text{Final BW }\left(g\right)-\text{Initial BW }\left(g\right), \end{array}$$

  $$\begin{array}{c} \text{SGR }\left(\text{\% }/d\right)=\frac{\left[\text{ LN }\left(\text{Final BW},g\right)-\text{LN }\left(\text{Initial BW},g\right)\;\right]}{\text{Experimental days }\left(d\right)}\times 100, \end{array}$$

  $$\begin{array}{c} \text{FI }\left(g/d\right)=\frac{\text{Amount of feed intake }\left(g\right)}{\text{Experimental days }\left(d\right)}, \end{array}$$

  $$\begin{array}{c} \text{FCR}=\frac{\text{Amount of feed intake }\left(g\right)}{\text{Total final BW }\left(g\right)-\text{Total initial BW }\left(g\right)}. \end{array}$$

The proximate composition of the fish body was determined as described above for the proximate analysis of diets.

### 2.4. RNA Extraction, Complementary DNA (cDNA) Synthesis, and Quantitative Real-Time Polymerase Chain Reaction (qPCR) Analysis

Head kidney, spleen, and mid-intestine from fish in each treatment (*n* = 3) were collected at the end of the feeding trial and preserved in NucleoProtect RNA (Macherey-Nagel, Germany), incubated at 4 °C for 24 h, and stored at −20 °C until RNA extraction. Total RNA was extracted from the tissue samples homogenized via liquid nitrogen grinding using the Monarch Total RNA Miniprep Kit (New England Biolabs, USA) in accordance with the manufacturer's protocol, with a final elution at 30 µL. The concentration of RNA was determined using a Biophotometer (D30, Eppendorf, Germany), and purity was evaluated by measuring the OD260/280 and OD260/230 absorption ratios. RNA integrity was verified by running 5 µL RNA on an ethidium bromide-stained 1% agarose gel submerged in 1× Tris Acetate EDTA (TAE) buffer, and the results were visualized using a gel documenter (Gel Doc XR+, Bio-Rad, USA). RNA was preserved at −80 °C for later use. cDNA was synthesized from 9 µL of RNA using a RevertAid RT Reverse Transcription Kit (Thermo Scientific, USA) according to the manufacturer's instructions. Mastercycler nexus gradient (Eppendorf, Germany) was used to run the cDNA synthesis with the following parameters of 25 °C for 5 min, followed by 42 °C for 60 min, and 70 °C for 5 min. The synthesized cDNA was preserved at −20 °C until it was used for qPCR analysis.

TaqMan-based qPCR was performed to determine the target gene expression between interleukin (IL)-1 beta (*IL-1β*), tumor necrosis factor alpha (*TNF-α*), *IL-6*, *IL-8*, and *IL-10*. The StepOne Real-Time PCR system (Applied Biosystems, USA) used in this experiment could run duplex probe reactions; hence, this experiment was designed using two probes, FAM and HEX, for all reactions. The primers and probes were designed by Apical Scientific Sdn. Bhd. (Selangor, Malaysia), and the relative information is listed in Table 2. The cDNA was diluted to a concentration of 13.5 ng/µL with a final volume of 10 µL, and then the reaction master mix was prepared. Each reaction comprised of 10 µL of SensiFAST Probe MasterMix, Hi-ROX (Bioline, UK), 7 µL of ddH_2_O, 1 µL of primer/probe mix 1, 1 µL of primer/probe mix 2, and 1 µL of cDNA samples. The thermal profile for all reactions was 2 min at 95 °C, followed by 40 cycles of 10 s at 95 °C, and 35 s at 60 °C. The expression levels of the target genes were calculated using the 2^−ΔΔCt^ method, and the data were normalized with β-actin as the housekeeping gene. Each sample assay was performed in triplicate.

### 2.5. In Vivo Bacterial Challenge

The bacteria employed in this study, *Vibrio parahaemolyticus*, was isolated from diseased Pacific white shrimp (*Litopenaeus vannamei*) cephalothorax collected from aquaculture farms and provided by the Laboratory of Marine Biotechnology, Institute of Bioscience, Universiti Putra Malaysia. The pathogenic strain was further confirmed using polymerase chain reaction (PCR) (with universal primers 27F and 1492R, 27F: 5′-AGAGTTTGATCMTGGCTCAG-3′, 1492R: 5′-TACGGYTACCTTGTTACGACTT-3′). Following overnight incubation in 3% marine Luria broth at 30 °C, bacteria were harvested by centrifugation (×10,000 rpm) and resuspended in 0.8% physiological saline solution (PSS) in a 50-mL centrifuge tube. Before the bacterial challenge test, a preliminary experiment was conducted to determine the dose of the pathogenic bacteria (LD_50_ was 0.03 × 10^7^ CFU/g). For the vibrio-challenge test, 20 fish were selected randomly and retained in each tank at the end of the 56-day feeding trial. Subsequently, each fish was anesthetized using MS-222 and then subjected to an intraperitoneal injection of 0.3 mL of a pathogenic strain at a concentration of 1 × 10^7^ CFU/mL. On the third day, each tank was poured with 30 mL of the same concentration of bacteria. Fish were subjected to a 24-h fasting period before the bacterial challenge test and re-fed with the respective experimental diets after 12 h. A range of vibriosis symptoms, including the presence of a thick layer of mucus on the body surface, fin congestion, and skin and muscle tissue hemorrhage and ulceration, were observed and recorded daily for 7 days. The cumulative mortality rate (CMR) of the fish was recorded and calculated as follows [25]:

  $$\begin{array}{c} \text{CMR}=\frac{\text{Number of dead fish}}{\text{Initial number of fish}}\times 100. \end{array}$$

### 2.6. Histological Analysis of Liver and Intestinal Samples

Moribund fish in the challenge experiments were instantly collected for histological analysis. The liver and mid-intestine from each fish of the tanks (*n* = 3) were dissected, removed, and stored in 10% formalin solution for histological analysis. The tissues were dehydrated using a range of graded (80%, 90%, 95% and 100%) ethanol baths, equilibrated with xylene, and subsequently embedded in paraffin wax, sections with a thickness of 5 µm were prepared, whereby two slides were cut for each sample at different depths in the wax block using a rotary microtome (RM2235, Leica, Germany), and subsequently stained with hematoxylin eosin (HE) using conventional histological methods. Slides were examined and representative pictures were taken under a light microscope (E200MV, Nikon, Japan) at 400-fold magnification, which was equipped with a photomicrograph connected to a computer with SmartV digital imaging system software (Jiangsu Jieda Technology Development Co. Ltd, China).

Morphometric evaluation of the liver was performed following the methodology outlined by Caimi et al. [26] with minor adjustments. In brief, six images per liver section were used to assess the following histological alterations: vascular congestion (VC), cytoplasmic vacuolization (CV), necrotic tissue (NT), inflammatory cell infiltration (II), and nuclear displacement (ND). The assessment of observed findings in liver sections employed a 5-point semiquantitative scoring system, where 0 denotes no alterations, 1 indicates minimal histopathology present in less than 25% of the observed areas, 2 represents minor histopathology present in less than 50% of the areas, 3 signifies moderate histopathology present in less than 75% of the areas, and 4 indicates severe histopathology observed in more than 75% of the areas.

To measure intestinal mucosal morphology, 10 villi per intestinal section were randomly selected from a cross-section. Villus length (VL), villus width (VW), muscular thickness (MT), and the number and density of goblet cells (GCs) per villus were measured following the protocols outlined by Hisano et al. [27] and Wassef et al. [28]. The villus surface (VS) was calculated using the formula [29] as follows:

  $$\begin{array}{c} \text{VS}=\;2\pi \times \;\frac{\text{VW}}{2}\times \text{VL}. \end{array}$$

### 2.7. Statistical Analysis

All data were analyzed using SPSS version 23.0. Before initiating statistical analysis, normality and homogeneity of variance for all dependent variables were assessed using Shapiro–Wilk's and Levene's tests. The histopathological scores were analyzed by Kruskal–Wallis ANOVA, and the results are expressed as the mean. The other indices underwent one-way ANOVA, and the subsequent Tukey's test was employed for post hoc analysis. The results are shown as means ± SE. Significant differences were declared at *p* < 0.05.

## 3. Results

### 3.1. Growth Performance and Feed Efficiency

The growth performance and feed efficiency of *L. calcarifer* under different dietary treatments are detailed in Table 3. At the start of the feeding trial, initial BW, BL, and CF values exhibited no statistically significant differences (*p* > 0.05). During the feeding trial, the SR was 88.65%–96.25% and was unaffected by any dietary treatment. At the end of the feeding trial, the VSI was significantly (*p* < 0.05) decreased due to palm carotene supplementation (ITpc and ITggp groups) compared with the control group. No statistically significant differences (*p* > 0.05) were observed among all experimental groups in terms of final BW, BL, HSI, TWG, SGR, FI, and FCR. The final CF of the fish fed with the immunostimulant supplement was comparable (*p* > 0.05) to that of the control group.

### 3.2. Proximate Composition of the Fish Body

Proximate analysis of the fish body after the feeding trial is presented in Table 4. In comparison with the control group, the crude protein in the ITgi group and the moisture in the ITggp group showed a significant (*p* < 0.05) increase. No significant difference (*p* > 0.05) was found in crude fat, ash, and carbohydrate in all immunostimulant supplementation groups compared with the control group.

### 3.3. Postchallenge Cumulative Mortality

**Figure 1:**
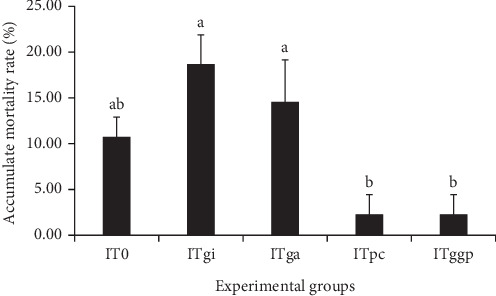
Effects of ginger, garlic, and palm carotene on the cumulative mortality rate (%) of *Lates calcarifer* challenged with *Vibrio parahaemolyticus* (mean ± SE, *n* = 3). Vertical bars assigned by different letters are significantly different (*p* < 0.05) and bars assigned the same letters do not have significant differences (*p* > 0.05). IT0: no immunostimulant, ITgi: incorporate ginger, ITga: incorporate garlic, ITpc: incorporate palm carotene, and ITggp: incorporate ginger + garlic + palm carotene.

The CMR of *L. calcarifer* after infection with *V. parahaemolyticus* is shown in Figure 1. The CMRs of the ITpc and ITggp groups were low at 2.22% compared with 10.69% in the control group. However, no statistical differences (*p* > 0.05) were found among the three aforementioned groups. In contrast, the CMRs of the ITgi (18.65%) and ITga (14.52%) groups were high and similar to those of control group (*p* > 0.05).

### 3.4. Histology of the Liver

**Figure 2:**
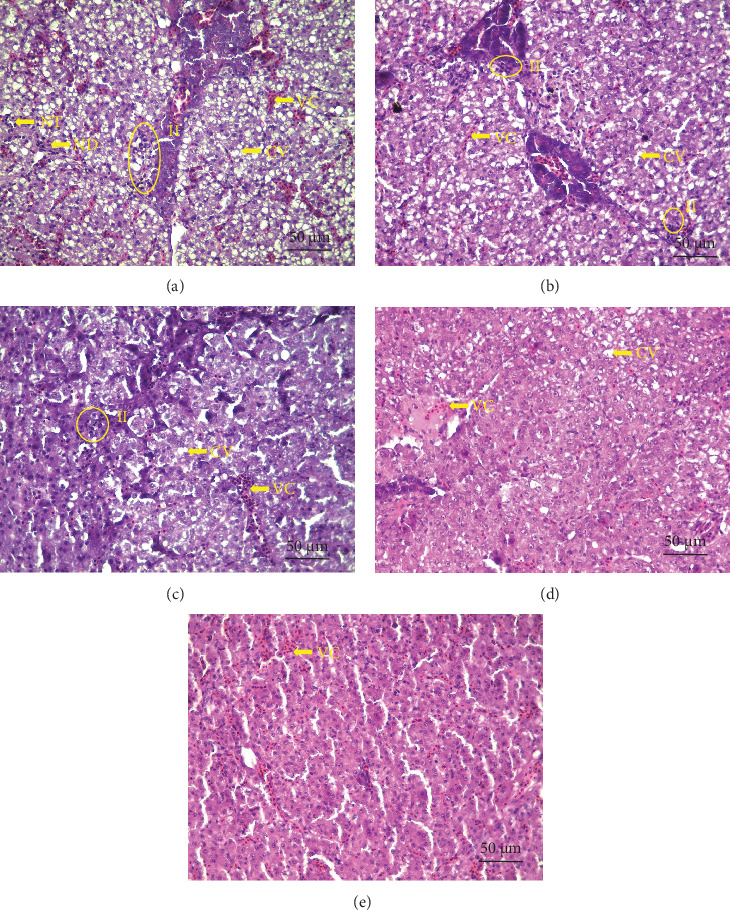
Representative micrographs of liver of *Lates calcarifer* challenged with *Vibrio parahaemolyticus* (HE stained, 400 × magnification, scale bar 50 µm). (A) IT0, (B) ITgi, (C) ITga, (D) ITpc, and (E) ITggp. CV, cytoplasm vacuolization; II, inflammatory-cell infiltration; ND, nuclear displacement; NT, necrotic tissue; VC, vascular congestion.

The histomorphological changes in the liver morphological examination of *L. calcarifer* challenged with *V. parahaemolyticus* are shown in Figure 2. Clear VC and CV with rare NT, II, and ND were observed. The histopathological assessment of the liver is shown in Table 5. In comparison to the control group, congestion scores exhibited a significant (*p* < 0.05) decrease with immunostimulant supplementation; the vacuolization score exhibited a significant (*p* < 0.05) increase in the ITpc group; necrosis scores and sum scores of pathological changes exhibited a significant (*p* < 0.05) decrease in the ITga, ITpc, and ITggp groups; and inflammation scores were significantly (*p* < 0.05) decreased in the ITpc and ITggp groups. However, the ND scores did not show significant differences (*p* > 0.05) across all experimental groups. Apart from the inflammation score, the poorest values of the other indices were recorded in the ITggp group.

### 3.5. Histology of the Intestine

**Figure 3:**
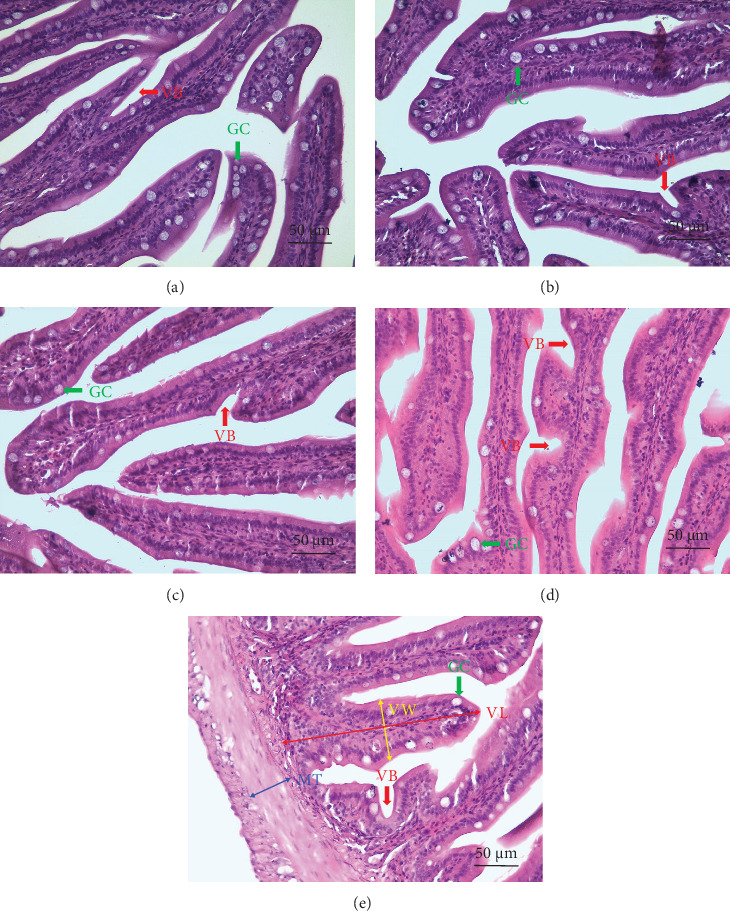
Representative micrographs of intestine of *Lates calcarifer* challenged with *V. parahaemolyticus* (HE stained, 400 × magnification, scale bar 50 µm). (A) IT0, (B) ITgi, (C) ITga, (D) ITpc, and (E) ITggp. The red arrow represents villus break (VB); the green arrow represents goblet cell (GC); the red double-headed arrow represents villus length (VL); the yellow double-headed arrow represents villus width (VW), which was measured at half of its length; the blue double-headed arrow represents muscular thickness (MT).

The histomorphological changes in the intestinal morphological examination of *L. calcarifer* challenged with *V. parahaemolyticus* are shown in Figure 3. Occasional instances of villus breakage and the presence of GC were observed in all experimental groups. Morphometric analysis of the intestinal mucosa is shown in Table 6. The ITggp group showed the highest values with statistical significance (*p* < 0.05) in VW, VS, and GC numbers per villus. Meanwhile, VS exhibited a significant (*p* < 0.05) increase in the ITpc group compared with the control group. However, the GC density exhibited a significant (*p* < 0.05) reduction in the ITgi group among treatments. No significant differences (*p* > 0.05) were noted in VL and MT.

### 3.6. Immune Gene Expression

**Figure 4:**
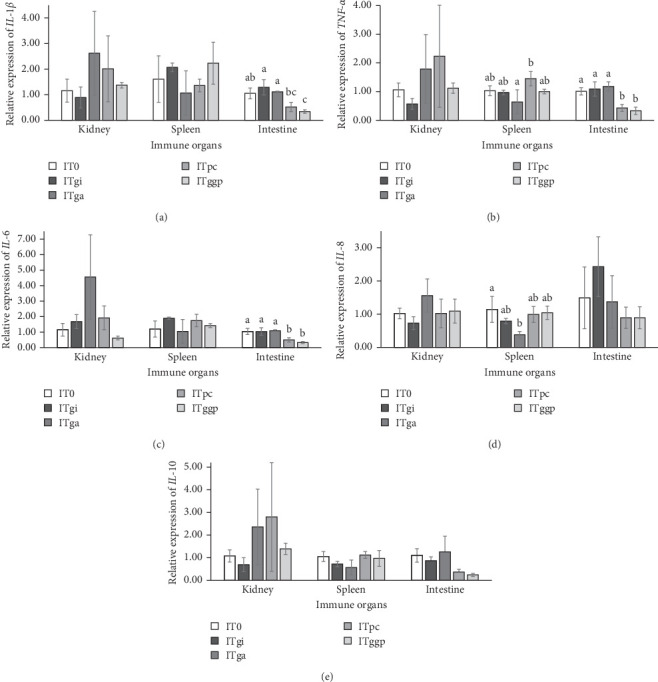
Effects of ginger, garlic, and palm carotene on the relative expression of interleukin (IL)-1 beta (*IL-1β*), tumor necrosis factor alpha (*TNF-α*), *IL-6*, *IL-8*, *IL-10* in the kidney, spleen, and intestine of *Lates calcarifer* (mean ± SE, *n* = 3). (A) *IL-1β*, (B) *TNF-α*, (C) *IL-6*, (D) *IL-8*, and (E) *IL-10*. Vertical bars assigned by different letters are significantly different, while bars assigned the same or no letters do not have significant differences among the experimental treatments in the same immune organ (*p* < 0.05).

The relative expression of *IL-1β*, *TNF-α*, *IL-6*, *IL-8*, and *IL-10* in the kidney, spleen, and intestine of *L. calcarifer* is shown in Figure 4. *IL-1β* expression in the intestine of the ITggp group, *TNF-α* and *IL-6* expression in the intestine of the ITpc and ITggp groups, and *IL-8* expression in the spleen of the ITga group were significantly (*p* < 0.05) downregulated compared with the control group. No significant difference (*p* > 0.05) was observed in the above-mentioned genes among other experimental groups in other organs. Furthermore, the relative expression of *IL-10* in all experimental groups was significantly unaffected (*p* > 0.05) by immunostimulant supplementation.

## 4. Discussion

Incorporation of immunostimulants in aquaculture has paved the way for advancements in fish health protection [30]. Many studies have proven that there are many types of immunostimulants that can activate the fish immune system. According to their sources, they can be roughly divided into saccharides and their complexes from microbes [31], animals [32], plants [33], organisms [34], and their extracts [35], chemical compounds [28], nutritional factors [36], hormones [37], and other cytokines [38]. Among them, plant immunostimulants have the advantages of abundant resources, low cost, drug residue-free, drug resistance-free, and convenient use, making them an important strategy for promoting the healthy and sustainable development of the aquaculture industry. Ginger, garlic, and palm carotene were chosen in the present study because of their availability, wide cultivation, and high production in Malaysia [39], especially palm oil, as the country holds a strategic position in the global production and export of palm oil. Biochemical conversion of palm oil solid waste into the extraction of carotene can promote faster growth in the sustainability of the industry [40]. There have been reports of supplementing the diet with ginger in *L. calcarifer* [8] and *O. niloticus* [9], garlic in *D. labrax* [13], and *C. gariepinus* [15, 16], β-carotene in *O. niloticus* [41] and *Piaractus mesopotamicus* [42]. Immunostimulants are crucial for enhancing nonspecific immune responses. For example, they can promote the synthesis of complement, lysozyme, protease inhibitor, C-reactive protein, natural hemolysin, agglutinin, α-macroglobulin, macrophage activating factor, and interferon [43]. They can activate the phagocytic and bactericidal functions of macrophages, neutrophils, and specific cytotoxic cells [44]. In addition, immunostimulants can improve the level of fish IgM antibodies and enhance the level of fish-specific immune responses [45]. Among the immunostimulants used in the present study, ginger and garlic are used worldwide for different chronic diseases, β-carotene is generally treated as an antioxidant in human and animal food/feed additives. The results indicated that the supplement of ginger, garlic, and palm carotene had almost no effect on the proximate composition of the feed, body composition, growth performance, and feed utilization of *L. calcarifer*. However, the VSI of the ITpc and ITggp groups decreased, indicating that palm carotene may have a positive effect on improving the health and reducing the inflammatory status of fish visceral organs.

*Vibrio parahaemolyticus* is a gram-negative pathogen that widely exists in aquaculture. These bacteria infect various aquatic animals, such as fish, shrimp, and shellfish. It is one of the main pathogens of aquatic animals [46]. The bacterial challenge test can directly and effectively evaluate the disease resistance of fish [47]. Our results indicate that palm carotene supplementation could enhance the *V. parahaemolyticus* resistance of *L. calcarifer*. Palm oil is renowned as one of the most abundant natural sources of plant-derived β-carotene, a vital nutrient essential for maintaining health [48]. β-carotene possesses potent antioxidant, anticancer, and anti-inflammatory properties [49]. The application of this substance in aquaculture feed has been reported in numerous papers and patents. For instance, dietary β-carotene supplement increased immune gene expression and immune-oxidative stress biomarkers of *O. niloticus* [50], improved antioxidant status, reduced harmful effects induced by cold in pacu (*Piaractus mesopotamicus*) [42, 51], and enhanced the mucosal immune responses of platy fish (*Xiphophorus maculatus*) [52]. Thus, β-carotene as a feed additive can improve the overall health of fish [53]. However, ginger and garlic alone supplements may not have exhibited positive effects on the disease resistance of *L. calcarifer* in the current study. This finding is incongruent with the observed results of *L. calcarifer* in the same percentage of ginger diet infected with *V. harveyi* [8] and olive flounder (*Paralichthys olivaceus*) in the same percentage of garlic extract diet challenged with *V. anguillarum* [54]. The disease protection could be attributed to the major pharmacologically active component of ginger, gingerol [55], and a complex of various allyl-containing sulfides, allicin, in garlic [56]. This study is an improvement based on our previous research on substituting fishmeal with BSFL meal, and 10% BSFL is included in each group (data unpublished). Antimicrobial peptides (AMPs), chitin, lauric acid, and other antibacterial substances from BSFL are effective immunostimulants for fish and shellfish. However, dietary BSFL and ginger or garlic did not show a synergistic effect on the disease resistance of *L. calcarifer* challenged with *V. parahaemolyticus* in the present study. Further research is needed to evaluate the long-term effects of BSFL and immunostimulants combination on the disease resistance of *L. calcarifer*. It has been reported that the antibacterial effect of ginger extract decreases in acidic environments or at temperatures above 80 °C [57], and garlic extract is prone to oxidation and has poor antibacterial ability when the temperature is above 50 °C [58]. Hence, exploring other feed production and preservation regimes should be considered.

Histology stands out as the gold standard for morphological analysis because it enables the study of substantial biological sections, allowing for the examination of the internal architecture of diverse tissues and cells. Limited information is currently available regarding the effects of immunostimulants on the histomorphology of the liver and intestine in bacteria-infected fish. The present study evaluated the liver and intestinal protection effects of ginger, garlic, and palm carotene by observing the histomorphological changes in *L. calcarifer* juveniles challenged with *V. parahaemolyticus*. The liver is an important target organ when infected with *V. parahaemolyticus* [59]. In this study, typical symptoms of bacterial septicemia gradually occurred in *L. calcarifer* after 12 h of infection with *V. parahaemolyticus*, such as organ bleeding and edema, hydropic degeneration, and VC. Inflammation was observed in liver HE-stained sections. This aligns with the pathological alterations observed in zebrafish (*Danio rerio*) challenged with *V. parahaemolyticus* [60]. It is noteworthy that ginger, garlic, and palm carotene all exhibit a certain degree of liver protection. Some studies have found that plant essential oils and many Chinese medicinal herbs have shown strong antimicrobial effects against *V. parahaemolyticus* by disrupting cell walls and membranes, interfering with cellular energy metabolism, and causing DNA loss or denaturation [61–63]. This may also be the antibacterial mechanism of ginger, garlic, and palm carotene in the present study. The NT and II in the ITpc and ITggp groups decreased, further supporting the view based on the VSI that palm carotene has an anti-inflammatory effect. However, the increased CV in the ITpc group may be a noteworthy side effect. At the same time, the NT in the ITga group also decreased, where the CMR increased. This suggests that the reduction of necrotic cells may not be the only factor determining mortality, and other factors, such as the expression of proinflammatory cytokines, may also play an important role.

The intestine is essential for the digestion and absorption of food and is considered a primary portal of entry for pathogens, given its direct exposure to the external environment [64]. In the current study, histological analyses did not reveal any indications of severe intestinal inflammation in any of the experimental groups. The lack of intestinal inflammation observed could be attributed to the fatty acid composition of BSFL (there was 10% BSFL inclusion in each treatment). BSFL is rich in saturated fatty acids (SFAs) with a notable concentration of lauric acid (C12). Many reports have shown its potential in improving intestinal health, attributed to its anti-inflammatory, antibacterial, and antiviral activities within the intestine [65]. However, high levels of inclusion of BSFL in fish feed may be indicative of liver lipid accumulation, potentially leading to the induction of intestinal inflammation. This agrees with previous reports of *L. calcarifer* [66], largemouth seabass (*Micropterus salmoides*) [67], Thai climbing perch (*Anabas testudineus*) [68], grass carp (*Ctenopharyngodon idellus*) [69], and zebrafish (*Danio rerio*) [70] fed BSFL meal. Hence, the high level of SFA in BSFL poses a limiting factor for their inclusion in feed. Among all experimental groups in this study, the combination of ginger, garlic, and palm carotene exhibited significant protective effects on the intestine. This evidence was shown in an increase in VW, VS, and the number of GCs in this treatment group. The intestinal villus correlates with an improvement in the digestion and absorption of nutrients. An increase in the number of GC in the intestine is typically linked to an immune response during the inflammation process [28]. In contrast, the decrease in GC density in the ITgi group resulted in a weakened intestinal barrier function, which may be related to an increased CMR.

BSFL has high fat content, leading to potential inflammation. To comprehensively evaluate the effect of immunostimulants on this potential inflammation, pro and anti-inflammatory genes were selected in this study. The cytokine *IL-1β* is a proinflammatory cytokine that plays a vital role in host immunological reactions against microbial infections, leading to proliferation of leucocytes and mediating the secretion of other cytokines. *TNF-α* exerts not only a cytotoxic effect on tumor cells and engages in a variety of pathophysiological processes, including viral and bacterial resistance, coagulation disorders, fever, inflammation, shock, multiorgan dysfunction, and formation of malignant fluid [71]. *IL-6* is recognized to be important to hematopoiesis and to have both pro and anti-inflammatory properties. It fosters the advancement of disease or aids in sustaining immunological responses [72]. Palm carotene supplementation led to a significant downregulation in the intestinal expression of pro-inflammatory cytokines *IL-1β*, *TNF-α*, and *IL-6* in this study. This supported the histological results in the intestinal morphological examination of *L. calcarifer* challenged with *V. parahaemolyticus*. These findings indicate that palm carotene may alleviate inflammation by regulating the intestinal immune response. *IL-8* serves as a chemokine that mobilizes neutrophils and is vital in orchestrating inflammatory reactions [73]. The relationship between the decrease in the *IL-8* expression in the spleen of the ITga group and the increase in the CMR is still unclear, and further research is needed to reveal the mechanism behind it. However, the relative expression of *IL-10* in all experimental groups was significantly unaffected by immunostimulant supplementation in this study. *IL-10* is an anti-inflammatory cytokine. It is the primary inhibitor of immune reactions and other factors [71]. This indicates that the anti-inflammatory effects of these immunostimulants on *L. calcarifer* are not dependent on the regulation of the *IL-10* pathway. These findings are inconsistent with the immune responses of *L. calcarifer* to dietary licorice (*Glycyrrhiza uralensis*) and probiotics (probiotic yeast *Saccharomyces cerevisiae* coupled with lactic acid bacteria *Lactobacillus casei*). In Yang et al.'s study [74], 1% dietary licorice upregulated the expression of *TNF*, *IL-8*, and *IL-10* in the kidneys of *L. calcarifer*. However, *IL-8* expression was significantly downregulated as the proportion of licorice increased to 3%, and *IL-10* expression was only downregulated when the licorice concentration reached 5%. *TNF* expression was always upregulated with an increase in licorice content. In Siddik et al. [75], significant upregulated expression of *TNF-α* and *IL-10* was observed in the intestine of probiotic-supplemented *L. calcarifer*. However, *IL-6* expression was unaffected by dietary probiotic supplementation. Little information is available about the effects of BSFL incorporated with immunostimulants on the gene expression of cytokines.

The downregulation of proinflammatory cytokine gene expression in this study may be due to immunostimulant supplementation effectively reducing the potential intestinal inflammation and liver damage caused by high levels of BSFL inclusion. To provide stronger support for the healthy development of the aquaculture industry, further research is required to explore the mechanisms, dose–response, and impacts on other physiological and immune indicators of BSFL incorporated with immunostimulants; further investigations are required to observe the long-term effects of BSFL incorporated with immunostimulants on the overall health of *L. calcarifer*.

## 5. Conclusion

The palm carotene supplements downregulated intestinal *TNF-α* and *IL-6* expression, decreased the sum scores of liver pathological changes and increased the intestinal VS compared with the control group. Moreover, the ginger, garlic, and palm carotene combined supplement show more promising immune gene expression (intestinal *IL-1β* was also downregulated compared to the control), liver and intestinal protection (lower sum scores of liver pathological changes and larger intestinal VS than palm carotene alone supplementation, GC number per intestinal villus was also increased compared to the control) effects on *L. calcarifer* infected with *Vibrio parahaemolyticus*. Therefore, it is recommended that *the L. calcarifer* diet could be supplemented with a combination of 0.5% ginger, 1% garlic, and 0.15% palm carotene in the feed formulation for sustainable *L. calcarifer* aquaculture industry.

## Figures and Tables

**Table 1:** Dietary formulation and proximate composition of the experimental diets for *Lates calcarifer*.

Items	Experimental diets				
	IT0	ITgi	ITga	ITpc	ITggp
Ingredients (%)					
Black soldier fly	10.0	10.0	10.0	10.0	10.0
Fishmeal	8.0	8.0	8.0	8.0	8.0
Corn gluten meal	8.0	8.0	8.0	8.0	8.0
Squid liver meal	2.5	2.5	2.5	2.5	2.5
Rice bran	7.0	7.0	7.0	7.0	7.0
Soybean meal	25.0	25.0	25.0	25.0	25.0
Fermented soybean meal	8.3	8.3	8.3	8.3	8.3
Wheat flour	22.4	21.9	21.4	22.25	20.75
Soybean oil	2.0	2.0	2.0	2.0	2.0
Lecithin oil	2.0	2.0	2.0	2.0	2.0
Monocalcium phosphate	1.5	1.5	1.5	1.5	1.5
Vitamin premix^a^	0.8	0.8	0.8	0.8	0.8
Mineral premix^b^	1.2	1.2	1.2	1.2	1.2
Lysine	0.4	0.4	0.4	0.4	0.4
Methionine	0.2	0.2	0.2	0.2	0.2
Choline chloride	0.3	0.3	0.3	0.3	0.3
Salt	0.4	0.4	0.4	0.4	0.4
Immune treatments (%)					
Ginger	0	0.5	0	0	0.5
Garlic	0	0	1.0	0	1.0
Palm carotene	0	0	0	0.15	0.15
Proximate composition (%, values expressed as mean, *n* = 2)					
Moisture	9.20	9.20	9.50	9.15	9.90
Protein	34.35	33.10	33.60	34.65	34.60
Fat	7.20	7.00	6.70	7.20	6.85
Ash	8.75	9.65	8.60	8.55	8.75
Carbohydrate	40.50	41.05	41.60	40.45	39.90

^a^1 kg of vitamin premix contained the following: VA 20.000 MIU, VD_3_ 4.000 MIU, VE 52.000 g, VK_3_ 4.000 g, VB_1_ 4.000 g, VB_2_ 10.000 g, VB_6_ 6.400 g, VB_12_ 40.000 g, pantothenic acid 22.40 g, nicotinic acid 80.00 g, folic acid 3.20 g, biotin 200.00 mg, phytase 10,000.00 FTU.

^b^1 kg of mineral premix contained the following: Zn 30.00 g, Mn 20.00 g, Fe 80.00 g, copper 4.000 g, iodine 1.500 g, selenium 0.125 g, cobalt 0.150 g, magnesium 50.00 g. IT0: no immunostimulant; ITgi: incorporate ginger; ITga: incorporate garlic; ITpc: incorporate palm carotene; ITggp: incorporate ginger + garlic + palm carotene.

**Table 2:** Primers and probes of immune genes for qPCR analysis.

Gene	Accession number	Band size (bp)	Primer sequence (5′–3′)	Probe sequence (5′–3′)
*β*-*actin* (*F*)	XM_018667666.2	115	F: CACCGCAAATGCTTCTAAACAG	CCAACCAAACGCCCAACAACTTCAGC
			R: CGCCTGAGTGTGTATGAGAAATG	

*IL-1β* (*H*)	XM_018669006.2	149	F: CTTCAGCACCCTCATGTCTG	ACAACAAGCCGGTGGAAATGTGCA
			R: CAAATACACTTCATGCCTGTGTC	

*TNF-α* (*F*)	XM_018695468.2	137	F: TGCCGTATATATGGGAGCTGTG	TGCTGCTTGATCTGGAGGACGAGC
			R: GCTTTCCTCACAAGGCAAACAC	

*IL-6* (*H*)	XM_018671215.2	148	F: TGTTCCAGCAGAAGGTCTATG	TGGTCCTGAAGACCTACAAAGAGCTGCT
			R: GCCTTAGGTCATCCTCAGAAG	

*IL-8* (*F*)	XM_018695863.2	110	F: GACACCTTGAAGAACCTTAACC	CCTACCCATAACAGGCAGGTCAGATGAA
			R: AAGTCTTTGTGCTCCATAAGTG	

*IL-10* (*H*)	XM_018686737.2	147	F: TCTTGAGCAAGATCACAACAAGAAG	TCCATCGAACCAGCAGCATCTGCA
			R: GAGACCGAGGAGTCATGCTG	

*Note: F* stands for FAM probe; *H* stands for HEX probe.

**Table 3:** Effects of ginger, garlic, and palm carotene on growth performance and feed efficiency of *Lates calcarifer* (mean ± SE, *n* = 3).

Parameters	Experimental groups				
	IT0	ITgi	ITga	ITpc	ITggp
Initial BW (g)	9.87 ± 0.13	10.06 ± 0.19	10.39 ± 0.18	9.61 ± 0.18	10.34 ± 0.25
Initial BL (cm)	7.86 ± 0.08	8.06 ± 0.10	8.12 ± 0.08	7.80 ± 0.09	8.02 ± 0.12
Initial CF (g/cm^3^)	2.03 ± 0.05	1.93 ± 0.04	1.94 ± 0.02	1.95 ± 0.03	1.99 ± 0.03
SR (%)	90.26 ± 1.17	91.35 ± 2.36	94.96 ± 3.41	96.25 ± 2.22	88.65 ± 2.90
Final BW (g)	23.85 ± 0.19	27.47 ± 3.09	25.93 ± 3.26	22.01 ± 2.25	26.24 ± 2.65
Final BL (cm)	10.41 ± 0.02	10.58 ± 0.50	10.46 ± 0.37	10.48 ± 0.74	10.87 ± 0.30
Final CF (g/cm^3^)	2.09 ± 0.08^ab^	2.23 ± 0.09^a^	2.25 ± 0.06^a^	1.91 ± 0.09^b^	2.03 ± 0.10^ab^
VSI (%)	9.18 ± 0.38^a^	9.01 ± 0.25^a^	8.48 ± 0.13^ab^	7.91 ± 0.27^b^	8.11 ± 0.19^b^
HSI (%)	2.01 ± 0.09	1.95 ± 0.06	1.83 ± 0.11	1.85 ± 0.11	2.03 ± 0.10
TWG (g)	13.98 ± 0.13	17.41 ± 2.92	15.54 ± 3.17	12.40 ± 2.26	15.90 ± 2.70
SGR (%/day)	1.57 ± 0.00	1.77 ± 0.19	1.61 ± 0.21	1.46 ± 0.20	1.64 ± 0.19
FI (g/day)	17.61 ± 0.13	18.04 ± 1.31	17.95 ± 1.10	17.28 ± 0.60	17.67 ± 0.66
FCR	2.75 ± 0.38	2.31 ± 0.15	2.23 ± 0.18	2.01 ± 0.09	2.38 ± 0.65

*Note:* Different superscript letters within a row indicate significant differences (*p* < 0.05); same or no superscript letters within a row indicate no significant differences (*p* > 0.05).

Abbreviations: BL, body length; BW, body weight; CF, condition factor; FCR, feed conversion ratio; FI, feed intake; HSI, hepatosomatic index; SGR, specific growth rate; SR, survival rate; TWG, total weight gain; VSI, viscerosomatic index.

**Table 4:** Effects of ginger, garlic, and palm carotene on body proximate composition (%) of *L. calcarifer* (mean ± SE, *n* = 3).

Ingredients	Experimental groups				
	IT0	ITgi	ITga	ITpc	ITggp
Moisture	73.83 ± 0.37^b^	72.90 ± 0.26^b^	73.63 ± 0.32^b^	74.23 ± 0.52^ab^	75.80 ± 1.15^a^
Protein	16.10 ± 0.93^b^	18.30 ± 0.47^a^	17.07 ± 0.47^ab^	17.37 ± 0.47^ab^	15.80 ± 0.74^b^
Fat	2.20 ± 0.25^ab^	2.57 ± 0.20^a^	2.17 ± 0.38^ab^	2.27 ± 0.23^ab^	1.63 ± 0.19^b^
Ash	4.93 ± 0.46	5.20 ± 0.42	5.13 ± 0.71	4.43 ± 0.32	4.90 ± 0.53
Carbohydrate	2.93 ± 0.86	1.37 ± 0.42	2.00 ± 1.12	2.40 ± 0.78	1.87 ± 0.57

*Note:* Different superscript letters within a row indicate significant differences (*p* < 0.05); same or no superscript letters within a row indicate no significant differences (*p* > 0.05).

**Table 5:** Effects of ginger, garlic, and palm carotene on liver histopathological assessment of *Lates calcarifer* (values expressed as mean, *n* = 3).

Items	Experimental groups				
	IT0	ITgi	ITga	ITpc	ITggp
Congestion	1.57^a^	1.19^b^	1.09^b^	1.21^b^	0.99^b^
Vacuolization	1.44^bc^	1.85^ab^	1.62^abc^	2.04^a^	1.21^c^
Necrosis	0.53^a^	0.44^ab^	0.31^b^	0.07^c^	0.03^c^
Inflammatory-cell infiltrate	0.82^a^	0.65^a^	0.72^a^	0.33^b^	0.45^b^
Nuclear displacement	0.37	0.27	0.32	0.25	0.25
Sum of pathological changes	4.72^a^	4.39^ab^	4.06^b^	3.89^b^	2.93^c^

*Note:* Different superscript letters within a row indicate significant differences (*p* < 0.05); same or no superscript letters within a row indicate no significant differences (*p* > 0.05).

**Table 6:** Effects of ginger, garlic, and palm carotene on intestinal histopathological measurement of *Lates calcarifer* (mean ± SE, *n* = 3).

Items	Experimental groups				
	IT0	ITgi	ITga	ITpc	ITggp
Villus length (μm)	55.48 ± 3.40^ab^	57.16 ± 2.23^ab^	54.71 ± 1.96^b^	61.85 ± 1.49^a^	60.76 ± 2.25^ab^
Villus width (μm)	7.56 ± 0.31^b^	8.44 ± 0.35^b^	7.69 ± 0.55^b^	8.23 ± 0.23^b^	9.86 ± 0.25^a^
Muscular thickness (μm)	18.41 ± 1.06	19.22 ± 1.19	18.84 ± 0.81	20.99 ± 1.08	20.34 ± 0.71
Villus surface (mm^2^)	1.34 ± 0.11^c^	1.53 ± 0.11^bc^	1.34 ± 0.13^bc^	1.60 ± 0.06^b^	1.88 ± 0.08^a^
Goblet cell number per villus	9.58 ± 0.91^bc^	6.95 ± 0.57^c^	8.60 ± 0.69^bc^	11.26 ± 0.87^b^	14.24 ± 1.27^a^
Goblet cell density (mm^2^)	7.28 ± 0.40^a^	4.57 ± 0.22^b^	6.72 ± 0.53^a^	6.98 ± 0.41^a^	7.56 ± 0.50^a^

*Note:* Different superscript letters within a row indicate significant differences (*p* < 0.05); same or no superscript letters within a row indicate no significant differences (*p* > 0.05).

## Data Availability

The data that support the findings of this study are available from the corresponding author upon reasonable request.

## References

[1] Nor N. M., Yazid S. H. M., Daud H. M., Azmai M. N. A., Mohamad N. (2019). Costs of Management Practices of Asian Seabass (*Lates calcarifer* Bloch, 1790) Cage Culture in Malaysia Using Stochastic Model That Includes Uncertainty in Mortality. *Aquaculture*.

[2] Future Market Insights (FMI), Asian Sea Bass Market. https://www.futuremarketinsights.com/reports/sea-bass-market.

[3] Shen X., Niu Y. C., Uichanco J. A. V. (2023). Mapping of a Major QTL for Increased Robustness and Detection of Genome Assembly Errors in Asian Seabass (*Lates Calcarifer*). *BMC Genomics*.

[4] Yamaguchi T., Quillet E., Boudinot P., Fischer U. (2019). What Could Be the Mechanisms of Immunological Memory in Fish. *Fish and Shellfish Immunology*.

[5] Zhao A., Xiao S., Kandelaki K. (2019). Knowledge, Perception, and Educational Status of Antimicrobial Resistance Among Chinese Medical Students. *Microbial Drug Resistance*.

[6] Vakaloloma U., Ho T. H., Loh J. Y. (2023). Modulation of Immune Genes in the Mucosal-Associated Lymphoid Tissues of Cobia by *Sarcodia Suae* Extract. *Veterinary Research Communications*.

[7] Annunziato G., Falavigna C., Pieroni M., Faccini A., Costantino G. (2018). *In Vitro* Digestion of *Zingiber officinale* Extract and Evaluation of Stability as a First Step to Determine Its Bioaccessibility. *Natural Product Communications*.

[8] Talpur A. D., Ikhwanuddin M., Bolong A. A. (2013). Nutritional Effects of Ginger (*Zingiber officinale* Roscoe) on Immune Response of Asian Sea Bass, *Lates calcarifer* (Bloch) and Disease Resistance Against *Vibrio Harveyi*. *Aquaculture*.

[9] Hassanin E. S., Elhakim Y. A. (2014). Dietary Effect of Ginger (*Zingiber officinale* Roscoe) on Growth Performance, Immune Response of Nile Tilapia (*Oreochromis niloticus*) and Disease Resistance Against, *Aeromonas hydrophila*. *Abbassa International Journal for Aquaculture*.

[10] Oghenochuko O. M., Mshelbwala F. M. (2021). Effects of *Moringa oleifera*, *Allium sativum*, *Zingiber Officinale* on the Haematological Parameters and Histopathological Changes in Visceral Organs of *Clarias gariepinus* Infected With, *Pseudomonas aeruginosa*. *ADAN Journal of Agriculture*.

[11] Labh S. N., Shakya S. R., Shakya R. (2014). Medicinal Uses of Garlic (*Allium sativum*) Improve Fish Health and Acts as an Immunostimulant in Aquaculture. *European Journal of Biotechnology and Bioscience*.

[12] Lee D. H., Ra C. S., Song Y. H., Sung K. I., Kim J. D. (2012). Effects of Dietary Garlic Extract on Growth, Feed Utilization and Whole-Body Composition of Juvenile Sterlet Sturgeon (*Acipenser ruthenus*). *Asian-Australasian Journal of Animal Sciences*.

[13] Irkin L. C., Yigit M., Yilmaz S., Maita M. (2014). Toxicological Evaluation of Dietary Garlic (*Allium sativum*) Powder in European Sea Bass *Dicentrarchus labrax* Juveniles. *Food and Nutrition Sciences*.

[14] Eirna-Liza N., Hassim H. A., Min C. C., Syukri F., Karim M. (2018). The Duration of Protection Conferred by Garlic on African Catfish (*Clarias gariepinus*) Against *Aeromonas hydrophila*. *Journal of Aquaculture Research and Development*.

[15] Agbebi O. T., Ogunmuyiwa T. G., Herbert S. M. (2013). Effect of Dietary Garlic Source on Feed Utilization, Growth and Histopathology of the African Catfish (*Clarias gariepinus*). *Journal of Agricultural Science*.

[16] Karim M., Eirna-liza N., Abu H., Hasliza S., Che R. (2016). The Effects of Dietary Inclusion of Garlic on Growth Performance and Disease Resistance of African Catfish (*Clarias gariepinus*) Fingerlings against *Aeromonas hydrophila* Infection. *Journal of Environmental Biology*.

[17] Suria K., Kulkarni A. D., Hazrulrizawati H., Yusoff M. M. (2015). Extraction of Palm Carotenes and Effect of Oxidative Degradation on *β*-Carotene. *International Journal of Engineering Research and Technology (IJERT)*.

[18] Yusoff M. Z. M. (2019). Loose Fruit Collector Machine in Malaysia: A Review. *International Journal of Engineering Technology and Sciences*.

[19] Garewal H. S., Ampel N. M., Watson R. R., Prabhala R. H., Dols C. L. (1992). A Preliminary Trial of *β*-Carotene in Subjects Infected With the Human Immunodeficiency Virus. *Journal of Nutrition*.

[20] Babin A., Saciat C., Teixeira M. (2015). Limiting Immunopathology: Interaction Between Carotenoids and Enzymatic Antioxidant Defences. *Developmental and Comparative Immunology*.

[21] Umegaki K., Uramoto H., Suzuki J., Esashi T. (1997). Feeding Mice Palm Carotene Prevents DNA Damage in Bone Marrow and Reduction of Peripheral Leukocyte Counts, and Enhances Survival Following X-Ray Irradiation. *Carcinogenesis*.

[22] Junichi O., Hoyoku N., Michiaki M. (1992). Palm Carotene Inhibits Tumor-Promoting Activity of Bile Acids and Intestinal Carcinogenesis. *Oncology*.

[23] Liu Y., Andin V. C., Chor W. K. (2024). A Preliminary Study on the Effects of Substituting Fishmeal With Defatted Black Soldier Fly (*Hermetia illucens*) Larval Meal on Asian Seabass (*Lates calcarifer*) Juveniles: Growth Performance, Feed Efficiency, Nutrient Composition, Disease Resistance, and Economic Returns. *Journal of Fish Biology*.

[24] Rahimnejad S., Lu K., Wang L. (2019). Replacement of Fish Meal With, *Bacillus Pumillus*, SE5 and, *Pseudozyma aphidis*, ZR1 Fermented Soybean Meal in Diets for Japanese Seabass (*Lateolabrax japonicus*). *Fish and Shellfish Immunology*.

[25] Xu F. M., Hou S. W., Wang G. X. (2021). Effects of Zymolytic Black Soldier Fly (*Hermetia illucens*) Pulp as Dietary Supplementation in Largemouth Bass (*Micropterus salmoides*). *Aquaculture Reports*.

[26] Caimi C., Gasco L., Biasato I. (2020). Could Dietary Black Soldier Fly Meal Inclusion Affect the Liver and Intestinal Histological Traits and the Oxidative Stress Biomarkers of Siberian Sturgeon (*Acipenser baerii*) Juveniles?. *Animals*.

[27] Huang B., Zhang S., Dong X. (2022). Effects of Fishmeal Replacement by Black Soldier Fly on Growth Performance, Digestive Enzyme Activity, Intestine Morphology, Intestinal Flora and Immune Response of Pearl Gentian Grouper (*Epinephelus fuscoguttatus* ♀ × *Epinephelus lanceolatus* ♂). *Fish and Shellfish Immunology*.

[28] Wassef E. A., Saleh N. E., Abdel-Meguid N. E., Barakat K. M., Abdel-Mohsen H. H., El-Bermawy N. M. (2020). Sodium Propionate as a Dietary Acidifier for European Seabass (*Dicentrarchus labrax*) Fry: Immune Competence, Gut Microbiome, and Intestinal Histology Benefits. *Aquaculture International*.

[29] Sakamoto K., Hirose H., Onizuka A. (2000). Quantitative Study of Changes in Intestinal Morphology and Mucus Gel on Total Parenteral Nutrition in Rats. *Journal of Surgical Research*.

[30] El-Gawad E. A. A., Asely A. M. E., Soror E. I., Abbass A. A., Austin B. (2020). Effect of Dietary *Moringa oleifera* Leaf on the Immune Response and Control of *Aeromonas hydrophila* Infection in Nile Tilapia (*Oreochromis niloticus*) Fry. *Aquaculture International*.

[31] dos Santos Voloski A. P., de Figueiredo Soveral L., Dazzi C. C., Sutili F., Frandoloso R., Kreutz L. C. (2019). *β*-Glucan Improves Wound Healing in Silver Catfish (*Rhamdia quelen*). *Fish and Shellfish Immunology*.

[32] Abdel-Tawwab M., Razek N. A., Abdel-Rahman A. M. (2019). Immunostimulatory Effect of Dietary Chitosan Nanoparticles on the Performance of Nile Tilapia, *Oreochromis niloticus* (L.). *Fish and Shellfish Immunology*.

[33] Stratev D., Zhelyazkov G., Noundou X. S., Krause R. W. M. (2018). Beneficial Effects of Medicinal Plants in Fish Diseases. *Aquaculture International*.

[34] Sánchez F., Lozano-Muoz I., Muoz S., Díaz N., Neira R., Wacyk J. (2023). Effect of Dietary Inclusion of Microalgae (*Nannochloropsis Gaditana* and *Schizochytrium* Spp) on Non-Specific Immunity and Erythrocyte Maturity in Atlantic Salmon Fingerlings. *Fish and Shellfish Immunology*.

[35] Mabrok Mahmoud, Abd Elaziz, Wahdan Ali (2018). The Immune Modulatory Effect of Oregano (*Origanum vulgare* L.) Essential Oil on *Tilapia zillii* Following Intraperitoneal Infection With *Vibrio anguillarum*. *Aquaculture International*.

[36] Jiang W. D., Zhou X. Q., Zhang L. (2019). Vitamin A Deficiency Impairs Intestinal Physical Barrier Function of Fish. *Fish and Shellfish Immunology*.

[37] Sanyal T., Sen K. (2018). Diversified Role of Prolactin in Fish: A Review. *Global Journal of Engineering Science and Researches*.

[38] Wen C. (2016). Immunoprotection of Crucian Carp With Recombinant Grass Carp (*Ctenopharyngodon idella*) Interferon Against Poly I: C Infection. *Fish and Shellfish Immunology*.

[39] Huang H. D. (2017). Overview of Agriculture in Malaysia. *World Tropical Agriculture Information*.

[40] Oseghale S. D., Mohamed A. F., Aja O. C. (2017). Status Evaluation of Palm Oil Waste Management Sustainability in Malaysia. *OIDA International Journal of Sustainable Development*.

[41] Hassaan M. S., Mohammady E. Y., Soaudy M. R., Sabae S. A., Mahmoud A. M. A., El-Haroun E. R. (2021). Comparative Study on the Effect of Dietary *β*-Carotene and Phycocyanin Extracted From Spirulina Platensis on Immune-Oxidative Stress Biomarkers, Genes Expression and Intestinal Enzymes, Serum Biochemical in Nile Tilapia, *Oreochromis niloticus*. *Fish and Shellfish Immunology*.

[42] Bacchetta C., Rossi A. S., Raú L. E., Cian R. E., Drago S. R., Cazenave J. (2019). Dietary *β*-Carotene Improves Growth Performance and Antioxidant Status of Juvenile *Piaractus mesopotamicus*. *Aquaculture Nutrition*.

[43] Srivastava P. K., Pandey A. K. (2015). Role of Immunostimulants in Immune Responses of Fish and Shellfish. *Biochemical and Cellular Archives*.

[44] Wang X. Q., Wei M. S., Wang J., Liu J., Zhang Q. B. (2023). Effects of Oligo-Porphyran on Immunological Parameters Related to Immunoregulation and Growth in RAW264.7 Macrophages and Zebrafish Model. *Aquaculture International*.

[45] Mehana E. E., Rahmani A. H., Aly S. M. (2015). Immunostimulants and Fish Culture: An Overview. *Annual Research and Review in Biology*.

[46] Ina-Salwany M. Y., Al-saari N., Mohamad A. (2019). *Vibriosis* in Fish: A Review on Disease Development and Prevention. *Journal of Aquatic Animal Health*.

[47] Wang M., Yi M. M., Lu M. X. (2019). Review on the Fish Health Assessment. *Acta Hydrobiologica Sinica*.

[48] Ghazali N. F., Hanim K. M., Pahlawi Q. A., Lim K. M. (2022). Enrichment of Carotene From Palm Oil by Organic Solvent Nanofiltration. *Journal of the American Oil Chemists’ Society*.

[49] Rohmah M., Rahmadi A., Raharjo S. (2022). Bioaccessibility and Antioxidant Activity of *β*-Carotene Loaded Nanostructured Lipid Carrier (NLC) from Binary Mixtures of Palm Stearin and Palm Olein. *Heliyon*.

[50] Taalab H. A., Mohammady E. Y., Hassan T. M. M., Abdella M. M., Hassaan M. S. (2022). *β*-Carotene of *Arthrospira Platensis* versus Vitamin C and Vitamin E as a Feed Supplement: Effects on Growth, Haemato-Biochemical, Immune-Oxidative Stress and Related Gene Expression of Nile Tilapia Fingerlings. *Aquaculture Research*.

[51] Bacchetta C., Ale A., Rossi A. S., Karakachoff M., Cazenave J. (2020). Effects of Cold Stress on Juvenile *Piaractus mesopotamicus* and the Mitigation by *β*-Carotene. *Journal of Thermal Biology*.

[52] Abdollahi Y., Ahmadifard N., Agh N., Rahmanifarah K., Hejazi M. A. (2019). *β*-Carotene-Enriched Artemia as a Natural Carotenoid Improved Skin Pigmentation and Enhanced the Mucus Immune Responses of Platyfish *Xiphophorus maculatus*. *Aquaculture International*.

[53] Yang J. J., Kim D. S. (2016). The Feed Additive for Abalone Including-Carotene KR20160103637A.

[54] Kim S. M., Jun L. J., Yeo I. K., Jeon Y. J., Jeong J. B. (2014). Effects of Dietary Supplementation With Garlic Extract on Immune Responses and Diseases Resistance of Olive Flounder, *Paralichthys olivaceus*. *Journal of Fish Pathology*.

[55] Jiang C. X., Lin L. Y., Song J., Cheng J. G. (2015). Research Progress on Gingerol and Zingiberol from Ginger. *Chinese Traditional and Herbal Drugs*.

[56] Shang A., Cao S. Y., Xu X. Y., Gan R. Y., Li H. B. (2019). Bioactive Compounds and Biological Functions of Garlic (*Allium sativum* L.). *Foods*.

[57] Zhang Z., Ma G. Q., Wang H. X., Hou W. F., Zhou M. (2020). A Study on the Antibacterial Activity of the Extracts of, *Zingiber officinale*, Roscoe against *Vibrio parahaemolyticus*. *Journal of Wuhan Polytechnic University*.

[58] Ma Y., Zhu B. T., Wang T. (2017). A Study on the Antibacterial Effect of Allicin on *Vibrio parahaemolyticus*. *Chinese J of Pub Health and Prev Med*.

[59] Peng W., Shi Y., Li G. F. (2016). *Tetraodon nigroviridis*: A Model of *Vibrio parahaemolyticus* Infection. *Fish and Shellfish Immunology*.

[60] Zhang W. M., Dong Q. H., Chen B., Zhang Y. H., Zu Y., Li W. M. (2016). Zebrafish as a Useful Model for Zoonotic *Vibrio parahaemolyticus* Pathogenicity in Fish and Human. Developmental and Comparative Immunology: Ontogeny, Phylogeny, Aging. *The Official Journal of the International Society of Developmental and Comparative Immunology*.

[61] Liu H. F., Wang F., Zhang S. Q., Li C. G., Ma Y. W. (2022). Effect of Chinese Herbal Compound on Immune Protection of Rainbow Trout (*Oncorhynchus mykiss*). *Journal of Guangdong Ocean University*.

[62] Yang W. Q., Qian L. L., Zhang Z. Y., Liu Y., Guo L. (2022). Identification of the Active Components of *Coptis chinensis* Extracts and Its Antibacterial Mechanism on *Vibrio parahaemolyticus*. *China Food Additives*.

[63] Sun Y., Wang X. D., Zhu J. L., Lu H. X., Ni Q. X. (2023). Antibacterial Activity and Mechanism of Lemon Grass Essential Oil (LG-EO) on *Vibrio parahaemolyticus*. *Journal of Chinese Institute of Food Science and Technology*.

[64] Rivadeneyra N. L. S., Mertins O., Cuadros R. C., Malta J. C. O., Matos L. V. D., Mathews P. D. (2020). Histopathology Associated With Infection by Procamallanus (Spirocamallanus) Inopinatus (Nematoda) in Farmed *Brycon cephalus* (Characiformes) from Peru: A Potential Fish Health Problem. *Aquaculture International*.

[65] Arturo V. A. J., Randazzo B., Foddai M. (2019). Insect Meal-Based Diets for Clownfish: Biometric, Histological, Spectroscopic, Biochemical and Molecular Implications. *Aquaculture*.

[66] Gupta S. K., Fotedar R., Foysal M. (2020). Impact of Varied Combinatorial Mixture of Non-Fishmeal Ingredients on Growth, Metabolism, Immunity and Gut Microbiota of *Lates calcarifer* (Bloch, 1790) Fry. *Scientific Reports*.

[67] Fischer H., Romano N., Renukdas N., Kumar V., Sinha A. K. (2022). Comparing Black Soldier Fly (*Hermetia illucens*) Larvae versus Prepupae in the Diets of Largemouth Bass, *Micropterus salmoides*: Effects on Their Growth, Biochemical Composition, Histopathology, and Gene Expression. *Aquaculture*.

[68] Mapanao R., Jiwyam W., Nithikulworawong N., Weeplian T. (2021). Effects of Black Soldier Fly (*Hermatia Illucens*) Larvae as a Fish Meal Replacement on Growth Performance, Feed Utilization, Morphological Characters and Carcass Composition of Thai Climbing Perch (*Anabas testudineus*). *Journal of Applied Aquaculture*.

[69] Lu R., Chen Y., Yu W. (2020). Defatted Black Soldier Fly (*Hermetia illucens*) Larvae Meal Can Replace Soybean Meal in Juvenile Grass Carp (*Ctenopharyngodon Idellus*) Diets. *Aquaculture Reports*.

[70] Zarantoniello M., Bruni L., Randazzo B. (2018). Partial Dietary Inclusion of *Hermetia illucens* (Black Soldier Fly) Full-Fat Prepupae in Zebrafish Feed: Biometric, Histological, Biochemical, and Molecular Implications. *Zebrafish*.

[71] Yi Y., Zhang Z., Zhao F. (2018). Probiotic Potential of *Bacillus velezensis* JW: Antimicrobial Activity against Fish Pathogenic Bacteria and Immune Enhancement Effects on *Carassius auratus*. *Fish and Shellfish Immunology*.

[72] Hunter C. A., Jones S. A. (2015). IL-6 as a Keystone Cytokine in Health and Disease. *Nature Immunology*.

[73] Kim K. H., Kim H. C., Park C. J., Park J. W., Lee Y. M., Kim W. J. (2019). Interleukin-8 (IL-8) Expression in the Olive Flounder (*Paralichthys olivaceus*) against Viral Hemorrhagic Septicemia Virus (VHSV) Challenge. *Development and Reproduction*.

[74] Yang R., Han M., Fu Z., Wang Y., Ma Z. (2020). Immune Responses of Asian Seabass *Lates calcarifer* to Dietary *Glycyrrhiza uralensis*. *Animals*.

[75] Siddik M. A. B., Foysal M. J., Fotedar R., Francis D. S., Gupta S. K. (2021). Probiotic Yeast *Saccharomyces cerevisiae* Coupled With *Lactobacillus casei* Modulates Physiological Performance and Promotes Gut Microbiota in Juvenile Barramundi, *Lates calcarifer*. *Aquaculture*.

